# Supplementary material for: Enhancement of phase transition temperature through hydrogen bond modification in molecular ferroelectrics
Source: Nat Commun. 2024 May 25;15:4470. doi: 10.1038/s41467-024-48948-0 (PMC11127950; doi:10.1038/s41467-024-48948-0)
Supplement: Supplementary file 1 — Supplementary Information [file 41467_2024_48948_MOESM1_ESM.pdf]

Supplementary Information

Enhancement of Phase Transition Temperature Through Hydrogen Bond Modification in Molecular Ferroelectrics

Yu-An Xiong[1‡], Sheng-Shun Duan[2‡], Hui-Hui Hu[1‡], Jie Yao[1], Qiang Pan[1], Tai-Ting Sha[1], Xiao Wei[2], Hao-Ran Ji[1], Jun Wu[2*], Yu-Meng You[1*]

[1]Jiangsu Key Laboratory for Science and Applications of Molecular Ferroelectrics, Southeast University, Nanjing 211189, People's Republic of China

[2]Joint International Research Laboratory of Information Display and Visualization, School of Electronic Science and Engineering, Southeast University, Nanjing 210096, People's Republic of China

‡Yu-An Xiong, Sheng-Shun Duan, Hui-Hui Hu contributed equally to this work

*Corresponding Author: youyumeng@seu.edu.cn; wujunseu@seu.edu.cn

Measurement Methods

Single-crystal X-ray crystallography

Single-crystal X-ray diffraction data of 1-hydroxy-3-adamantanammonium tetrafluoroborate [(HaaOH)BF_4] and 1-adamantanammonium tetrafluoroborate [(Haa)BF_4] were measured using a Rigaku Saturn 924 diffractometer with Mo-Kα radiation (λ = 0.71073 Å). Data collection, cell refinement, and data reduction was performed using Rigaku CrystalClear 1.3.5. The structures were solved by direct methods and refined by the full-matrix method based on F^2 using the SHELXTL software package. All non-hydrogen atoms were refined anisotropically and the positions of all hydrogen atoms were generated geometrically. The data collection and structure refinement of these crystals are summarized in Supplementary Table 1. The structures were deposited at the Cambridge Crystallographic Data Centre (CCDC: 2192545, 2192429, 2314039) and can be obtained free of charge from the CCDC via www.ccdc.cam.ac.uk/getstructures.

Powder X-ray diffraction

Powder X-ray diffraction (PXRD) data were measured using a Rigaku D/MAX 2000 PC X-ray diffraction system with Cu Kα radiation in the 2θ range of 5°–50° with a step size of 0.02° step size. The powder samples of (HaaOH)BF_4 and (Haa)BF_4 were placed in the sample bin.

Differential scanning calorimetry

Differential scanning calorimetry (DSC) measurements were performed on a Netzsch Model DSC 200 F3 instrument under N_2 and one atmospheric pressure, with a 20 K/min heating/cooling rate. The powder sample of about 10.0 mg was placed in the aluminum crucible.

Thermogravimetric analysis

Thermogravimetric analysis (TGA) was performed on the NETZSCH TG209 F3 apparatus with a 10 K/min heating rate under air atmosphere using the powder samples of (HaaOH)BF_4 and (Haa)BF_4.

Dielectric measurements

The samples were made with pressed-powder pellet and single crystals oriented perpendicular to the corresponding crystal axis, respectively. Silver conductive paste deposited on the plate surfaces was used as electrodes. Complex dielectric permittivities were measured with a TH2828A impedance analyzer over the frequency range from 500 Hz to 1 MHz with an applied electric field of 0.5 V.

Ferroelectric hysteresis loop measurements

The double-wave method was utilized to test the polarization-voltage curve on a homemade measuring system composed of a voltage source (Trek 609E-6), waveform generator (Keysight 33500B) and current meter (Keithley 6517B). Testing probes with indium gallium alloy were connected to different positions on the sample film, respectively, for collecting the in-plane current signals when triangular-wave voltage was applied. The polarization value was obtained by integrating the reversal current peak and dividing the area of liquid InGa alloy droplet.

Piezoresponse force microscopy characterization

The piezoresponse force microscopy (PFM) measurement was carried out on a commercial piezoresponse force microscope (Oxford instrument, MFP-3D) with high-voltage package and in-situ heating stage at 298 K. PFM is based on the atomic force microscopy (AFM), with an AC drive voltage applied to the conductive tip. Conductive Pt/Ir-coated silicon probes (EFM, Nanoworld) were used for domain imaging and polarization switching studies, with a nominal spring constant of ~2.8 nN/nm and a free-air resonance frequency of ~75 kHz. Since the amplitude of the low-frequency vertical PFM was within the noise level of the quadrant photodetector of the AFM, we performed the PFM experiments at contact resonance. The drive frequency was 355 kHz for out-of-plane PFM images and 660 kHz for in-plane PFM images.

Piezoelectric coefficient measurements

For macroscopic piezoelectric coefficient (d_{33}), we adopted the quasi-static method by a commercial piezometer (Piezotest, model: PM200). The single crystal plates for (Haa)BF_4 and (HaaOH)BF_4 were clamped between two flat metal plates, then a dynamic force of about 0.25 N along different axes was applied.

Preparation and measurements of piezoelectric energy-harvesting devices

All device fabrication processes were performed under ambient air and room-temperature conditions. The piezoelectric energy-harvesting devices consist of the top conductive adhesive tape, (HaaOH)BF_4, and the bottom conductive adhesive tape. The entire assembly is encapsulated using polydimethylsiloxane (PDMS) and cured at room temperature for one day. The formed piezoelectric devices were polarized under a direct current voltage. The preparation process for (Haa)BF_4 and blank devices is identical as well.

To measure the output performance, a fabricated device was placed on a custom-design mechanical system, where the device was periodically pressed by a shaker (active area 2×2 cm^2). The force applied on the device was instantly monitored by a force sensor (502F01, YMC) during the measurement. A digital oscilloscope (DSO4032A, Keysight) was used to record the output performance of devices

(Supplementary Fig. 26). To measure the sensor performance, a device was secured onto a dummy model. After grounding the upper electrode and connecting the upper and bottom electrodes to a digital oscilloscope (SDS6034H10Pro, SIGLENT), the output performance was tested by tapping and assessing its response.

Calculate condition

For the sake of further investigating the microscopic ferroelectric polarization, we carried out density functional calculations based on the Berry phase method developed by Kingsmith and Vanderbilt.[1, 2] The first-principles calculations were performed within the framework of density functional theory (DFT) implemented in the Vienna ab initio Simulation Package (VASP).[3, 4] The energy cutoff for the expansion of the wave functions was fixed at 500 eV, and the exchange–correlation interactions were treated within the generalized gradient approximation of the Perdew–Burke–Ernzerhof type.[5] For the integrations over the k-space, we applied a 3 × 3 × 5 k-point mesh.

Hershefield surface analysis

The molecular Hershefield surface in the crystal structure is constructed on the basis of the electron distribution of the sum of the electron densities of spherical atoms.[6] By defining the standard distance, the Hershefield surface of the molecule is derived from the Hershefield surface based on the standard distance function. The intermolecular interaction information in the crystal can be obtained by analyzing the Hershefield surface of the molecule in the crystal.[7] The nearest distance from the point on the Hershefeld surface to the inner and outer atoms of the surface is defined as d_i and d_e, respectively. The standard distance d_{norm} can be calculated using formula (1).[8]

$$d_{norm}=\frac{d_i-r_i^{vdW}}{r_i^{vdW}}+\frac{d_e-r_e^{vdW}}{r_e^{vdW}} \qquad (1)$$

In (1), r^{vdW} is the atomic van der Waals radius. The red part on the Hershefield surface represents that the calculated standard distance d_{norm} is less than the total van der Waals radius, the blue part represents that the calculated standard distance d_{norm} is greater than the total van der Waals radius, and the white part is between the two distances.

As a supplement to the Hershefield surface, 2D fingerprint can more intuitively display the crystal structure characteristics. The 2D fingerprint is formed by mapping

the points on the Hershefield surface with d_i as the abscissa and d_e as the ordinate.

Energy framework analysis

CrystalExplorer 17[9, 10] was used to evaluate and visualize the pairwise interaction energies of $(HaaOH)BF_4$ crystal. The calculations are based on the B3LYP/6-31G(d.p) molecular wavefunctions. The energy components calculated within this method are electrostatic, polarization, dispersion, and exchange-repulsion and finally the total interaction energy, where E_total = 1.057 E_ele + 0.740 E_pol + 0.871 E_disp + 0.618 E_rep. Pairwise interaction energies between molecules were calculated considering radius of 3.8 Å from centroid of a molecule to an atom (of another molecule) belonging to its nearest neighbor. The tube size used in all the energy frameworks was 300, and the lower energy threshold (cutoff) value was set to 1.

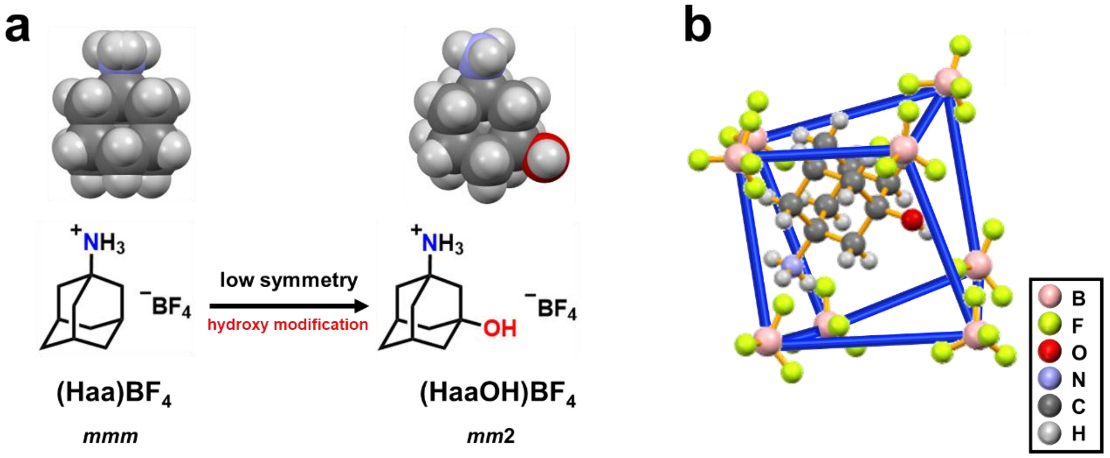

Supplementary Fig. 1 | Schematic diagram of hydrogen bond modification design strategy. (a) Design strategy of hydrogen bond modification in molecular ferroelectric $(HaaOH)BF_4$. (b) The twisted cage in the ferroelectric phase of $(HaaOH)BF_4$.

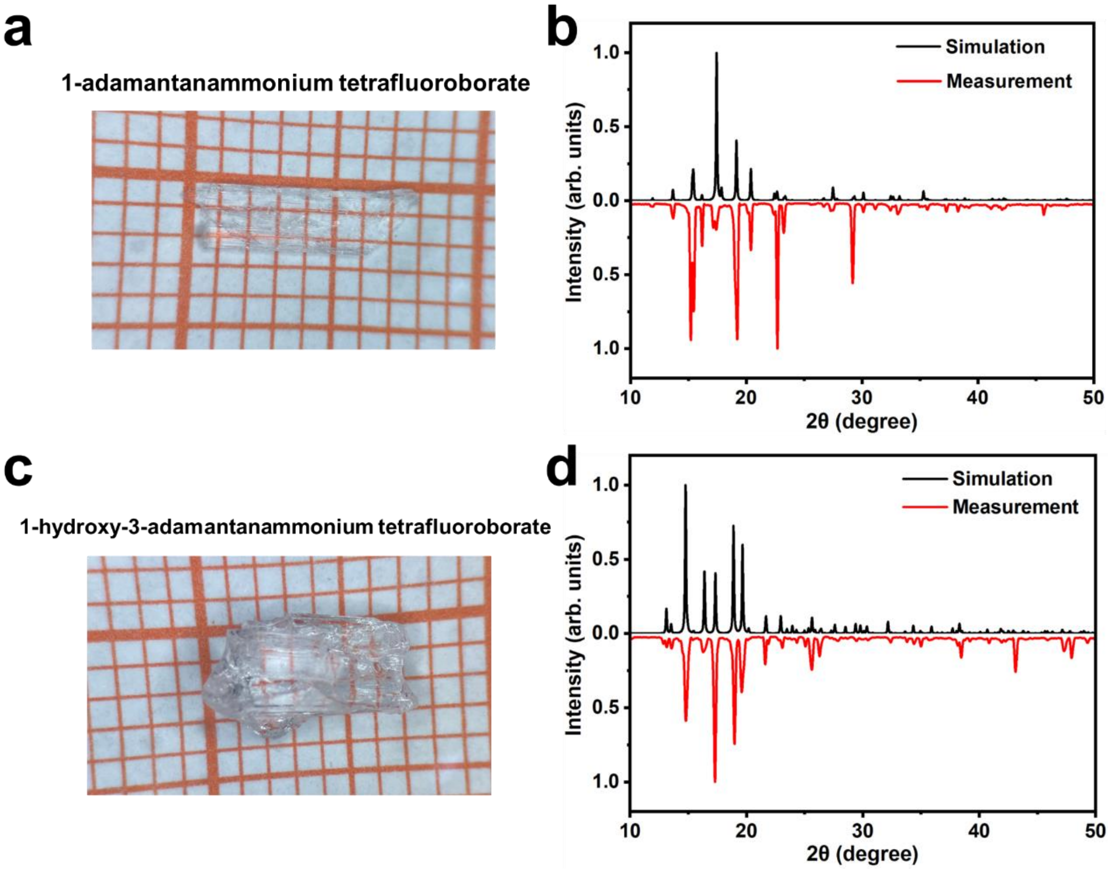

Supplementary Fig. 2 | The crystal photographs and the powder X-ray diffraction patterns. The crystal photographs of (a) (Haa)BF_4 and (c) (HaaOH)BF_4. The measured powder X-ray diffraction patterns of (b) (Haa)BF_4 and (d) (HaaOH)BF_4 at 293 K and the comparison with the simulation of their crystal data respectively.

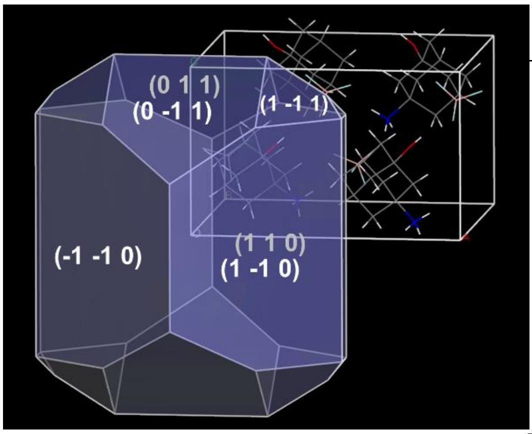

hkl	Distance	Total facet area %
{1 1 0}	11.37673000	55.67300987
{0 1 1}	14.82842000	13.54068182
{0 1 -1}	14.82842000	13.54068182
{2 0 0}	15.28677999	4.46769528
{1 1 1}	16.68242939	6.14730970
{1 1 -1}	16.68242939	6.14730970

Supplementary Fig. 3 | Morphology calculation of (HaaOH)BF_4 by BFDH.

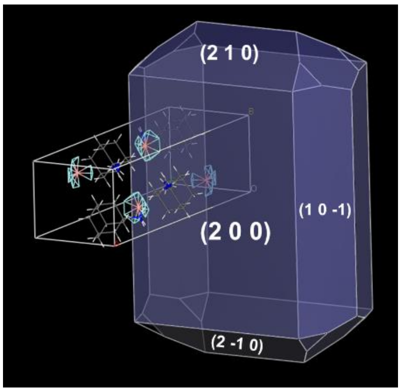

hkl	Distance	Total facet area %
{2 0 0}	8.71679989	38.61050242
{1 0 1}	13.47964131	30.90171757
{2 1 0}	17.34295217	21.67539231
{0 1 1}	19.68503161	6.81739078
{1 1 1}	20.16174892	1.99499692

Supplementary Fig. 4 | Morphology calculation of (Haa)BF_4 by BFDH.

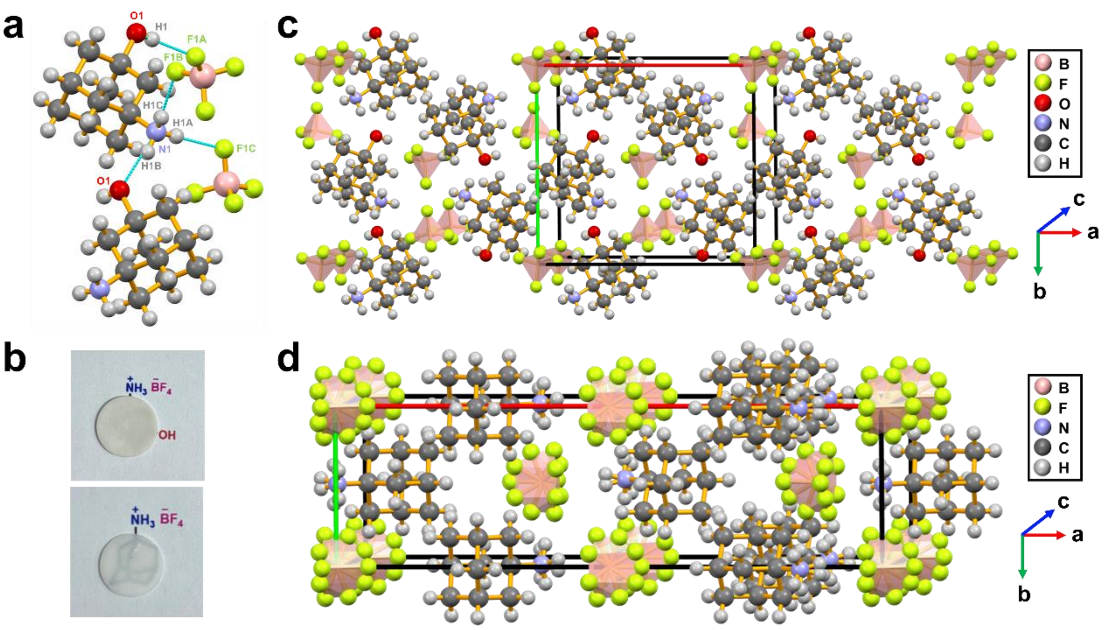

Supplementary Fig. 5 | Pressed powder and crystal structure diagrams. (a) The schematic diagram of hydrogen bonds in (HaaOH)BF_4. (b) Pressed powders of (HaaOH)BF_4 (top) and (Haa)BF_4 (bottom). Packing view of crystal structures of (c) (HaaOH)BF_4 and (d) (Haa)BF_4 at 293 K.

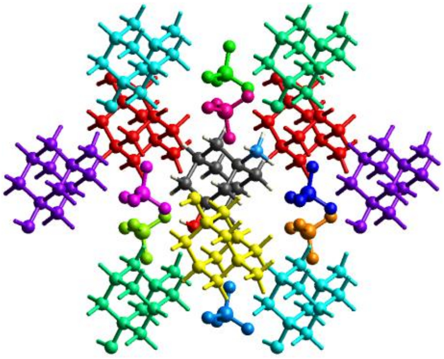

	N	Symop	R	Electron Density	E_ele	E_pol	E_dis	E_rep	E_tot
	2	-x, -y, z+1/2	7.24	B3LYP/6-31G(d,p)	-0.2	-0.2	-14.2	4.5	-9.9
	1	-	5.88	B3LYP/6-31G(d,p)	0.0	nan	0.0	0.0	nan
	2	x+1/2, -y+1/2, z	7.27	B3LYP/6-31G(d,p)	0.0	0.0	0.0	0.0	0.0
	1	-	5.57	B3LYP/6-31G(d,p)	-0.4	-0.1	-3.8	0.2	-3.7
	1	-	5.74	B3LYP/6-31G(d,p)	-0.2	-0.2	-14.2	4.5	-9.9
	2	-x+1/2, y+1/2, z+1/2	7.32	B3LYP/6-31G(d,p)	-39.3	-11.7	-19.5	121.5	7.8
	2	-x+1/2, y+1/2, z+1/2	7.32	B3LYP/6-31G(d,p)	-1.0	-0.2	-6.1	0.2	-6.4
	1	-	6.93	B3LYP/6-31G(d,p)	-0.3	-0.0	-0.3	0.0	-0.6
	1	-	5.56	B3LYP/6-31G(d,p)	0.3	-0.4	-9.0	1.2	-7.2
	2	x, y, z	8.20	B3LYP/6-31G(d,p)	-0.4	-0.1	-3.8	0.2	-3.7
	1	-	5.23	B3LYP/6-31G(d,p)	0.1	-0.0	-0.4	0.0	-0.2
	1	-	5.52	B3LYP/6-31G(d,p)	-1.0	-0.2	-6.1	0.2	-6.4

Supplementary Fig. 6 | Molecular structure pairs and the interaction energies

(kJ/mole) obtained from energy frameworks calculation for (HaaOH)BF_4.

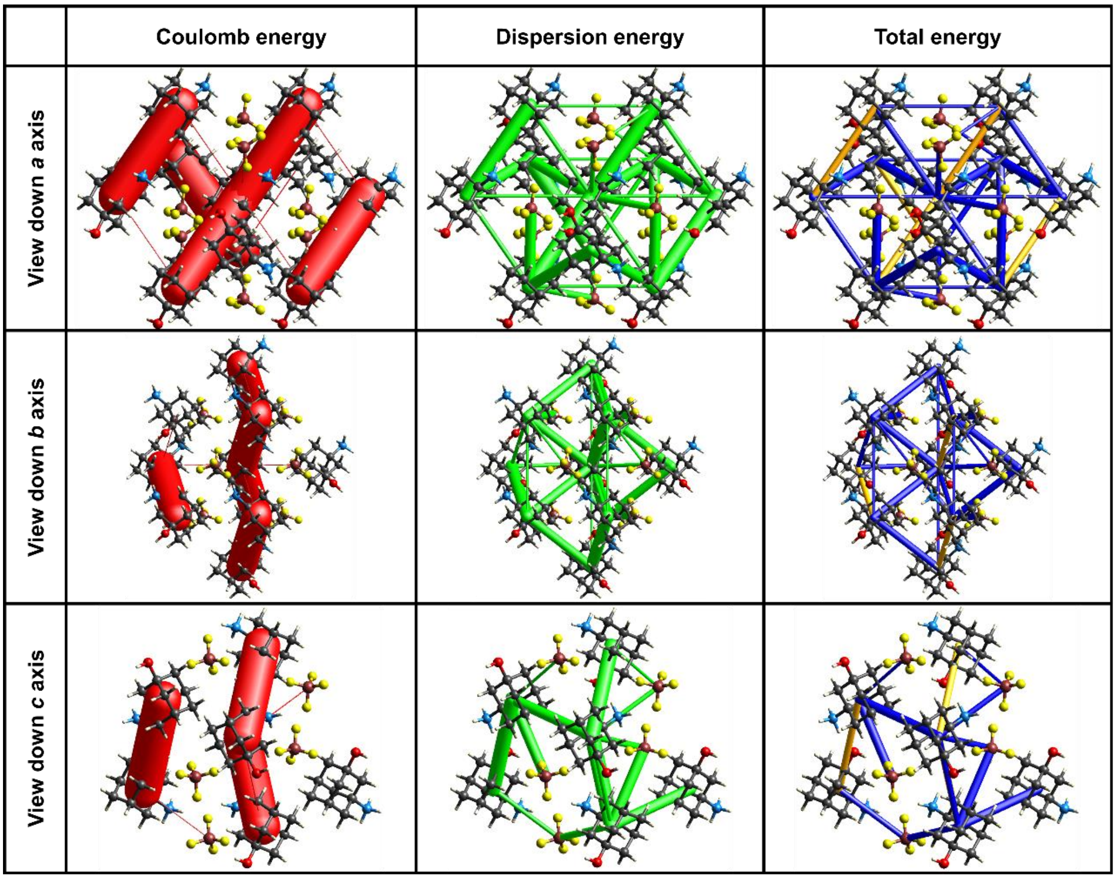

Supplementary Fig. 7 | Energy frameworks of (HaaOH)BF_4 viewed along different crystallographic directions.

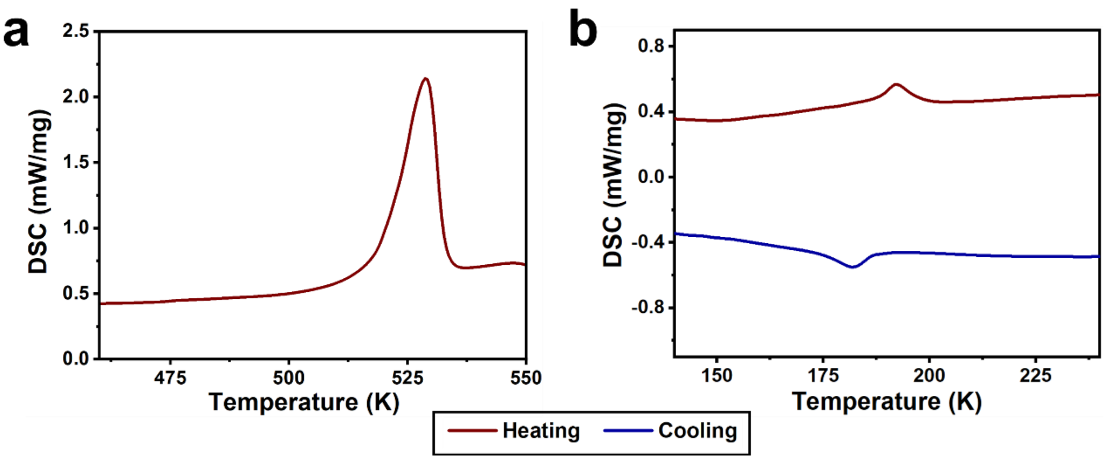

Supplementary Fig. 8 | DSC measurements of (HaaOH)BF_4 and (Haa)BF_4. DSC curves of (HaaOH)BF_4 (a) and (Haa)BF_4 (b). The red lines represent the heating process and the blue line represents the cooling process. The red line represents the heating

process, while the blue line represents the cooling process.

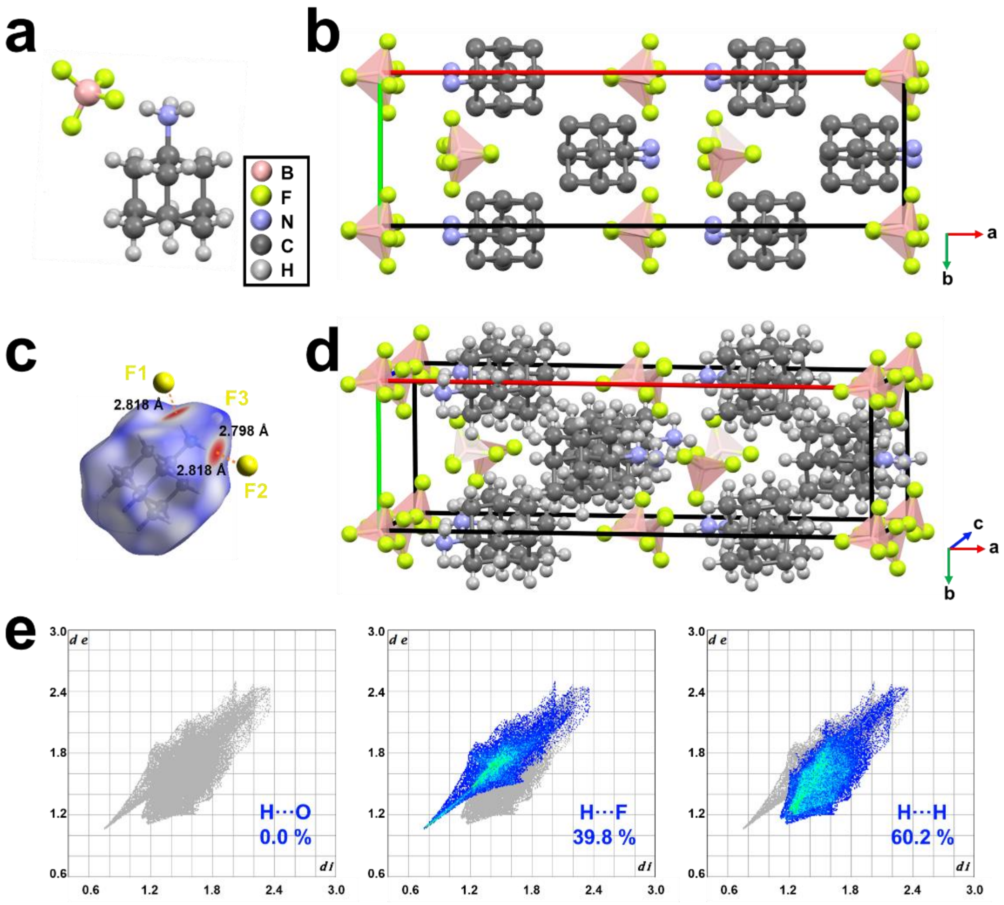

Supplementary Fig. 9 | Crystal structure of (Haa)BF_4 at 163 K. (a) Asymmetric units of (Haa)BF_4. (b) Packing view along the *c*-axis. The anions and cations are ordered. Parts of hydrogen atoms are omitted for clarity. (c) Hirshfeld surfaces of the guest cations in (Haa)BF_4. (d) Packing view of crystal structures of (Haa)BF_4. (e) Decomposed fingerprint plots and proportion for the H···O, H···F and H···H contacts of the guest cations in (Haa)BF_4 on the Hirshfeld surfaces are displayed respectively.

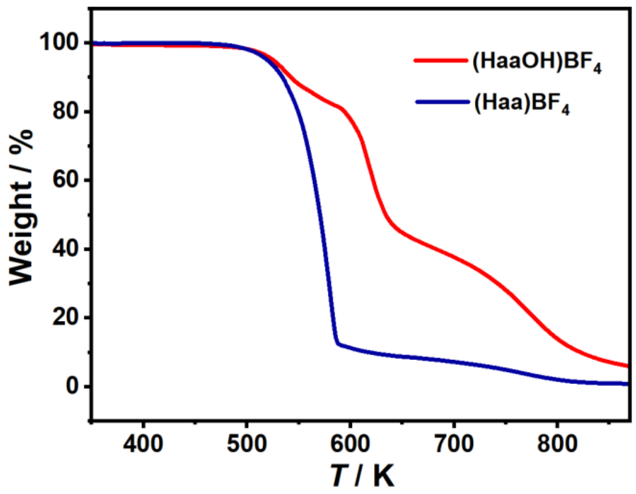

Supplementary Fig. 10 | TGA curve of (HaaOH)BF_4 (red) and (Haa)BF_4 (blue).

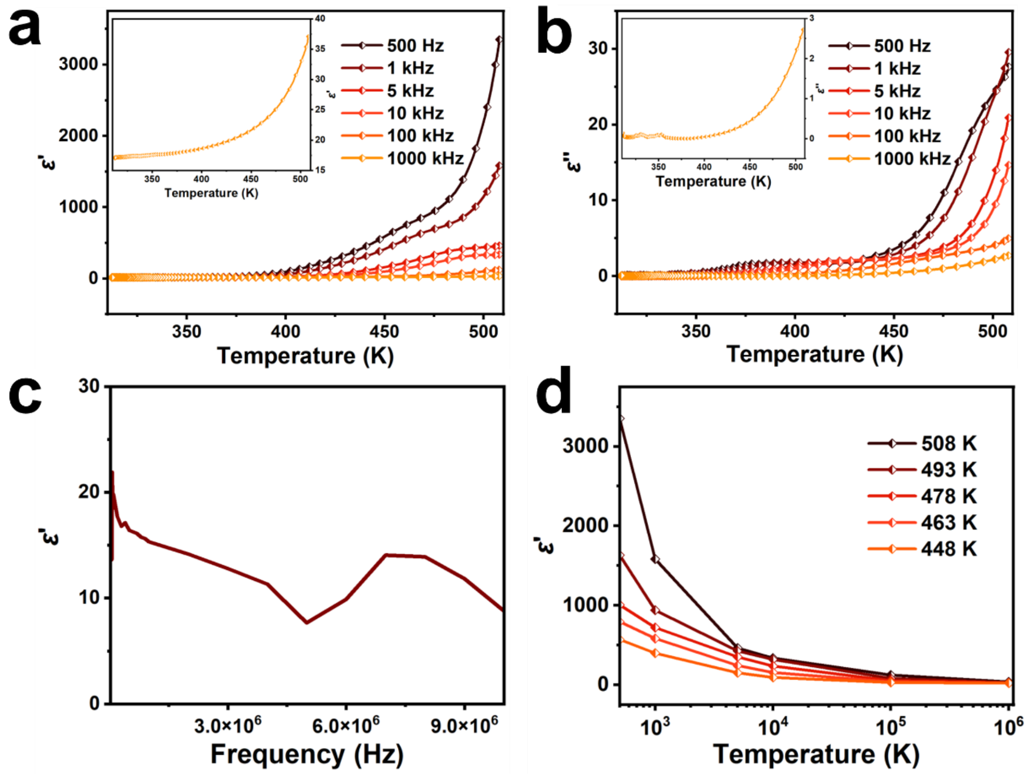

Supplementary Fig. 11 | Variable-temperature and variable-frequency complex dielectric constant of (HaaOH)BF_4 measured on the single-crystal along *c*-axis. Temperature-dependent dielectric constant of real part ε' (a) and imaginary part ε'' (b). Frequency-dependent dielectric at 293 K (c) and at different temperatures (d). The complex dielectric constant $\varepsilon = \varepsilon' - i\varepsilon''$, in which ε' and ε'' are the real and imaginary parts, respectively. The insets in (a) and (b) show the temperature-dependent ε' and ε'' at the frequency of 1000 kHz, respectively.

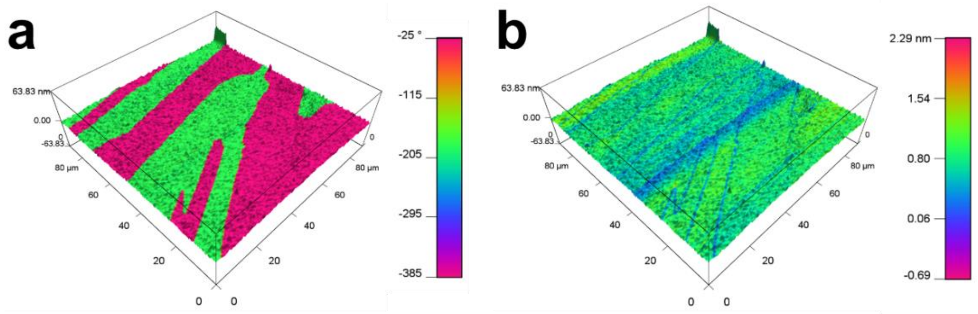

Supplementary Fig. 12 | As-grown domain structure of (HaaOH)BF_4 film. PFM phase (a) and amplitude (b) superimposed on the 3D topographic image for (HaaOH)BF_4 thin film measured at 298 K. The scan area is 90 μm × 90 μm.

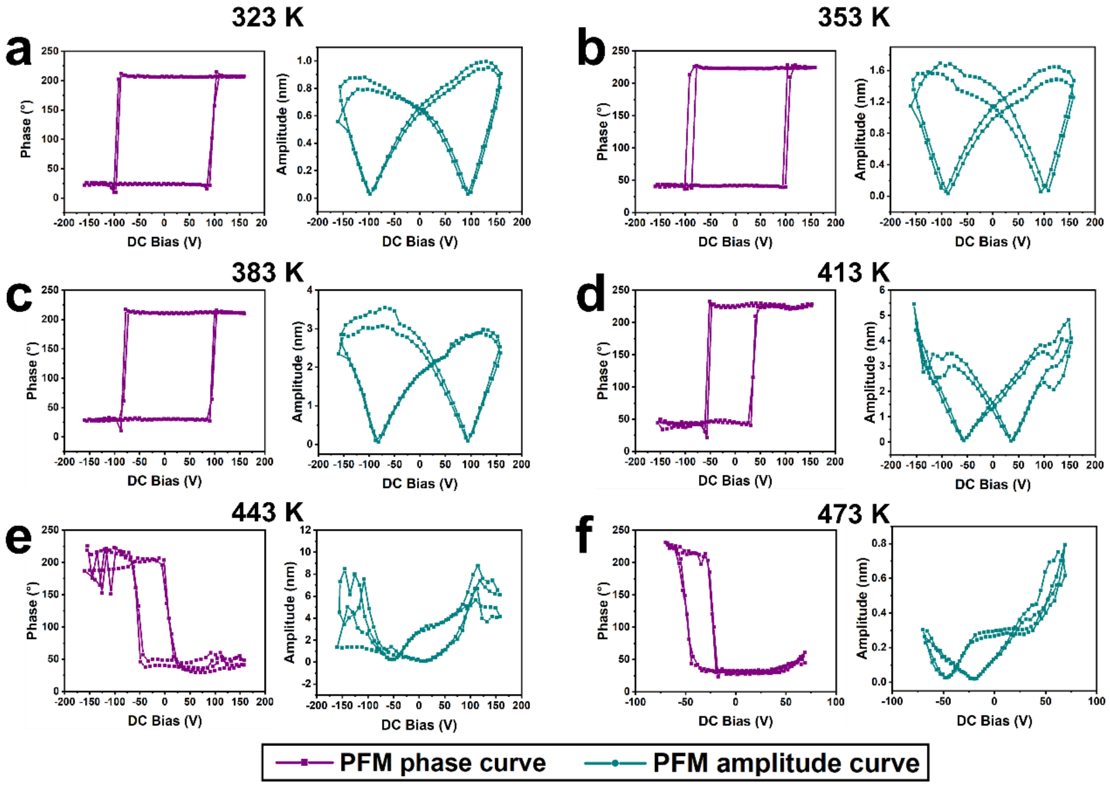

Supplementary Fig. 13 | Temperature-dependent switching spectroscopy PFM measurements. The PFM phase loops and the PFM amplitude curves at (a) 323 K, (b) 353 K, (c) 383 K, (d) 413 K, (e) 443 K and (f) 473 K. The PFM phase curves are represented in purple, and the PFM amplitude curves are represented in green.

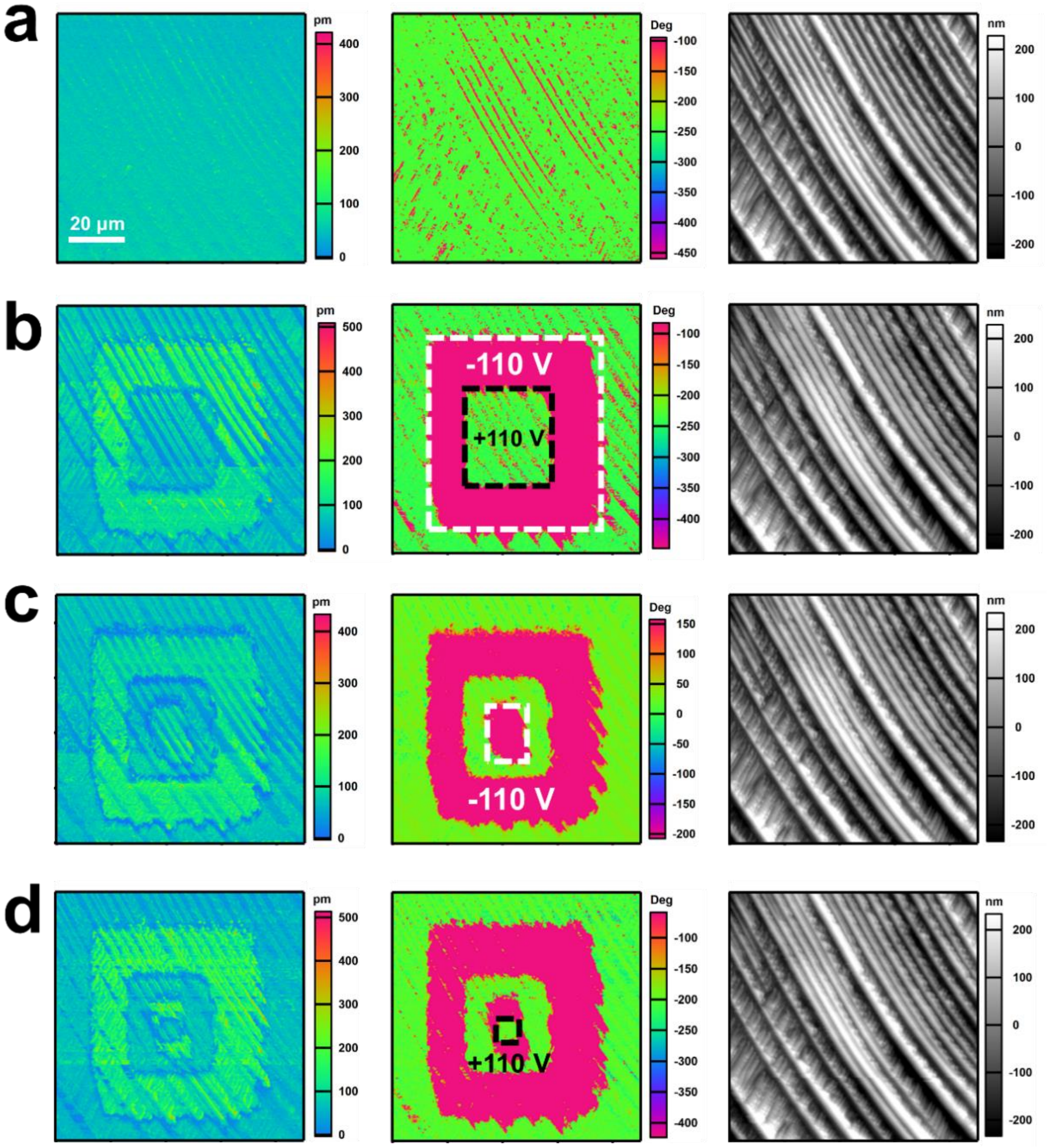

Supplementary Fig. 14 | Polarization switching behavior for (HaaOH)BF_4. PFM amplitude (left), PFM phase (middle) and topography (right) images for a selected region of the (HaaOH)BF_4 thin film (a) in the pristine state, (b) after electric poling on the white and black-box region with a tip bias of -110 V and +110 V, respectively. (c) The imaging of domain switching after electric poling on the white-box region with a tip bias of -110 V, and (d) after electric poling on the black-box region with a tip bias of +110 V.

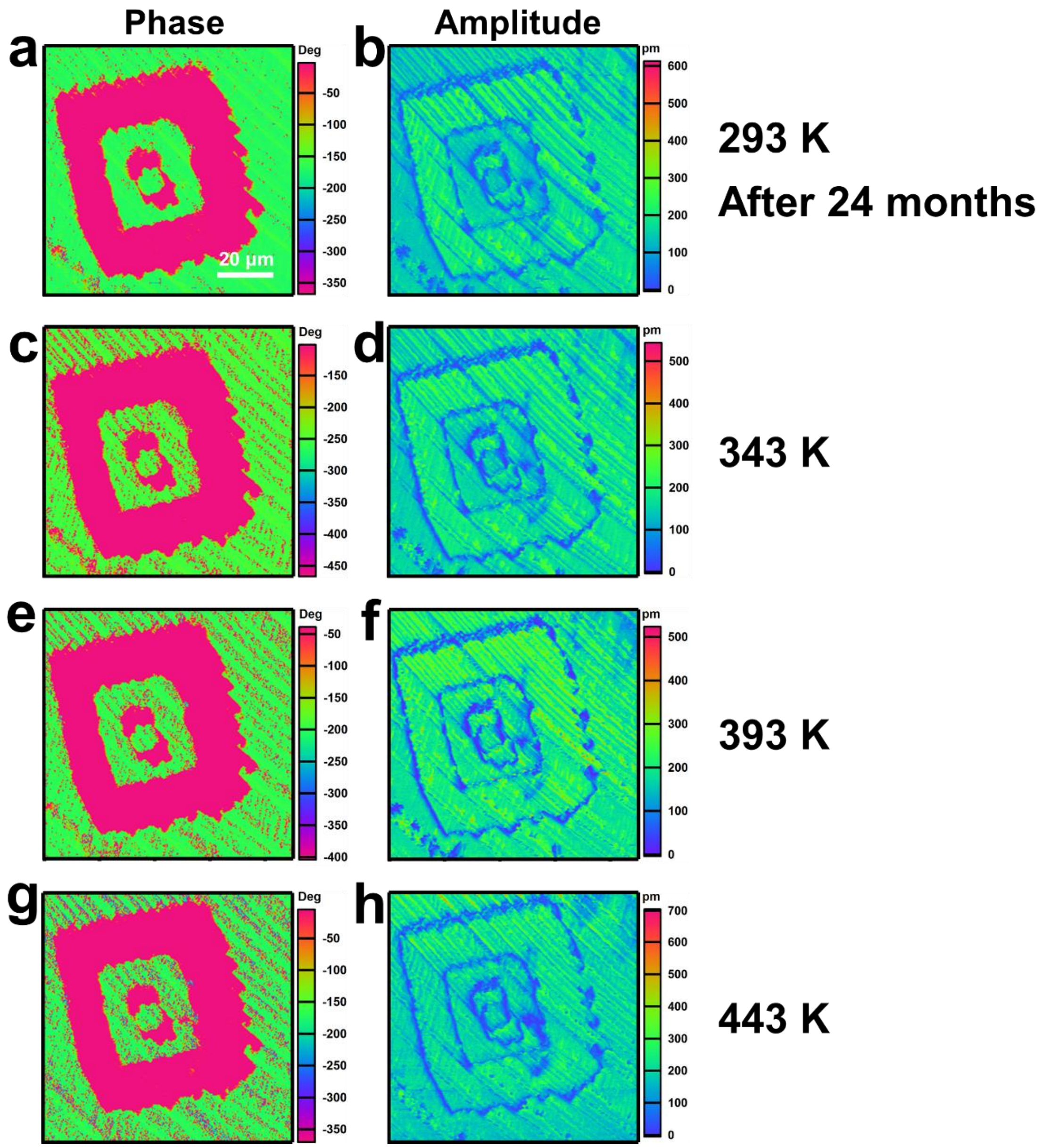

Supplementary Fig. 15 | Evolution of the domain structure of (HaaOH)BF_4 at various temperatures. The domain imaging after 24 months. Lateral PFM phase and amplitude images (a, b) at 293 K, (c, d) at 343 K, (e, f) at 393 K and (g, h) at 443 K, respectively.

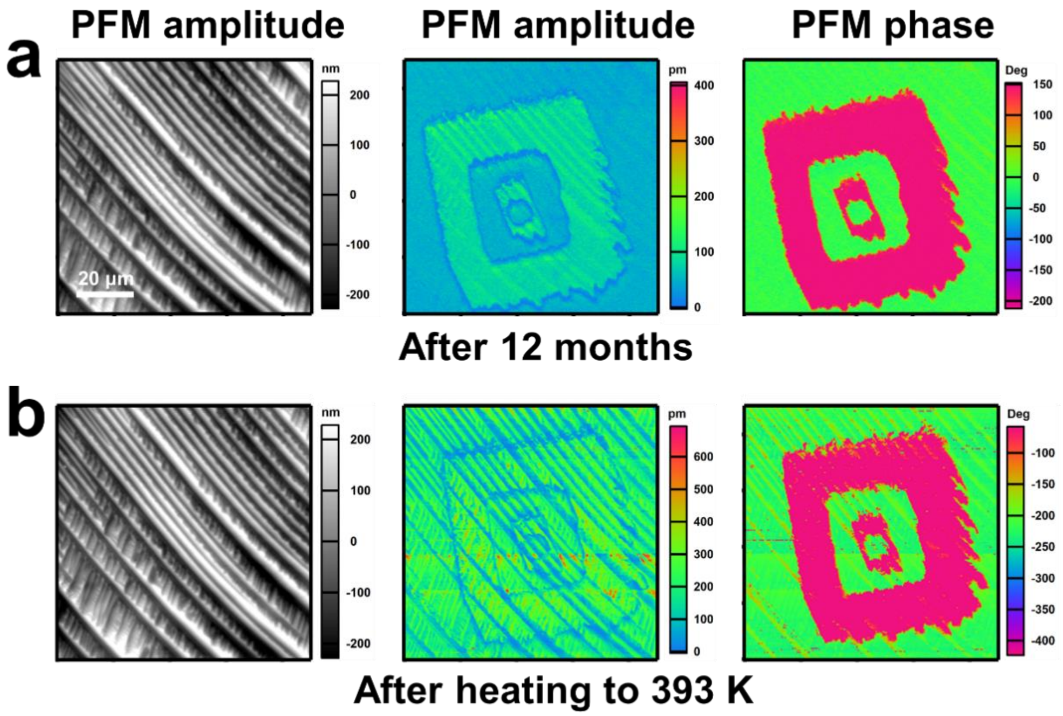

Supplementary Fig. 16 | The stability of domains written by the tip bias. (a) The domain imaging after 12 months. (b) The domain imaging after heating to 393 K.

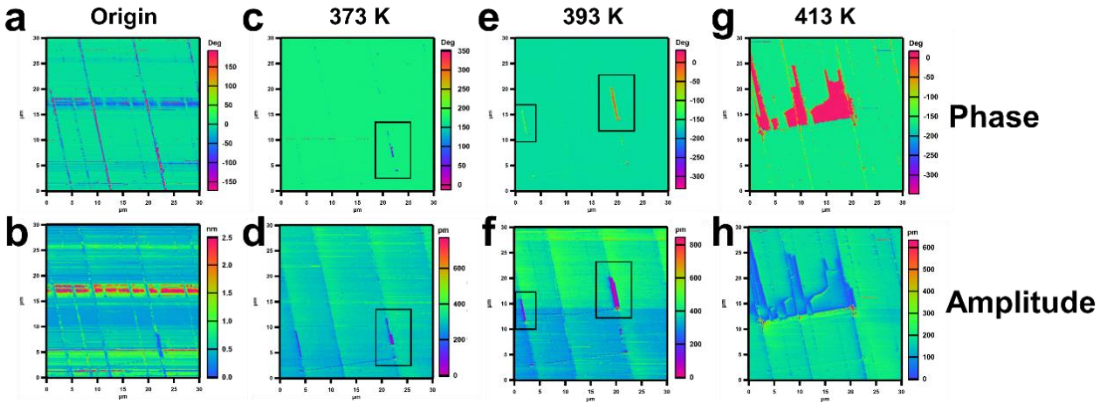

Supplementary Fig. 17 | Revolution of the domain at various temperature. The original PFM phase (a) and amplitude (b) at the selected 30 × 30 μm^2. The domain revolution under the same electric polarization of −160 V DC voltage at (c, d) 373 K, (e, f) 393 K and (g, h) 413 K.

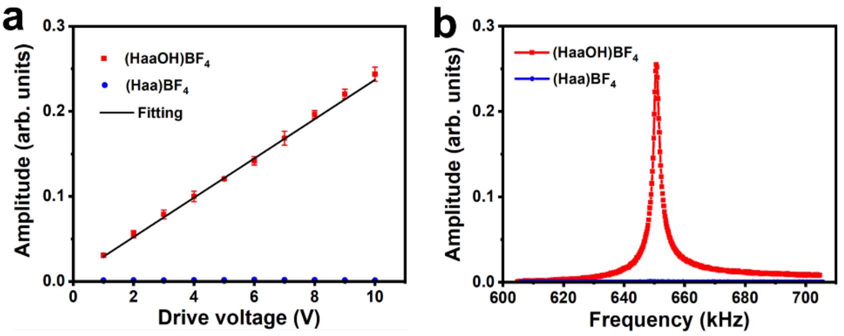

Supplementary Fig. 18 | PFM piezoresponse signal measurements. Comparison of PFM effective piezoelectric coefficient (a) and PFM resonance peaks (b) of $(HaaOH)BF_4$ and $(Haa)BF_4$ films. Data are presented as mean ± standard deviation of 3 data points on a single film.

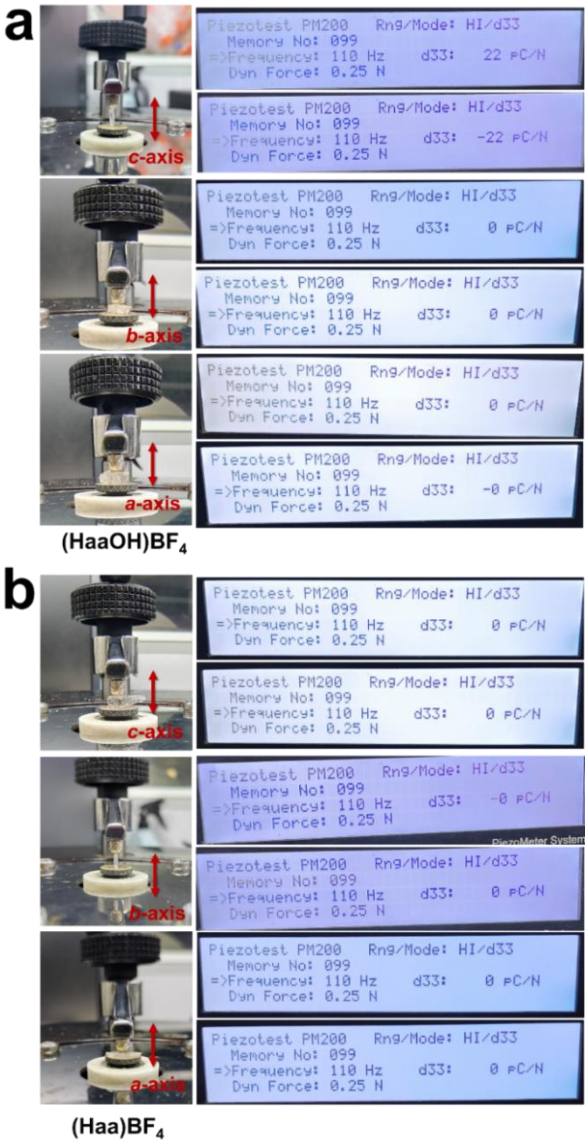

Supplementary Fig. 19 | Piezoelectric coefficient d_{33} measurements. Piezoelectric

coefficient d_{33} on (HaaOH)BF_4 (a) and (Haa)BF_4 (b) crystals using the quasi-static method along different axis directions.

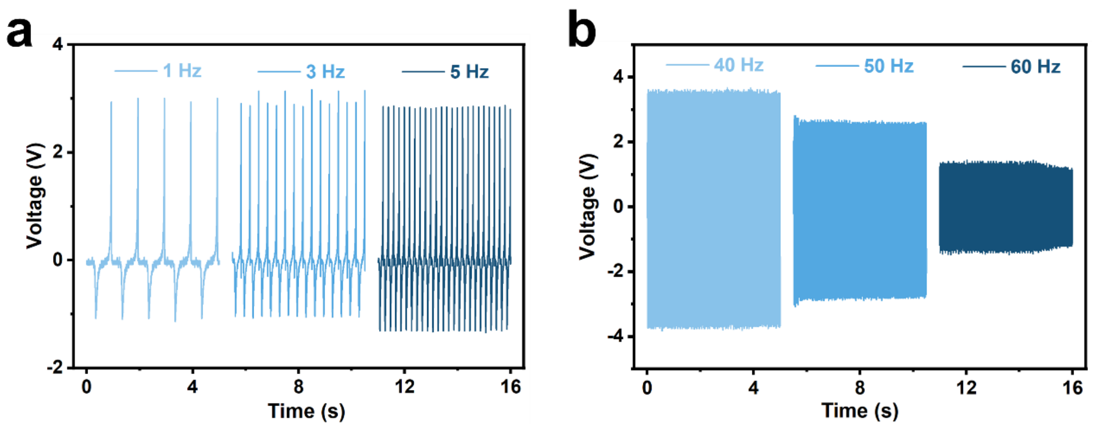

Supplementary Fig. 20 | The performance of the (HaaOH)BF_4 energy harvester at various force frequencies. (a) The response signals at low frequency of 1 Hz, 3 Hz and 5 Hz. (b) The response signals at high frequency of 40 Hz, 50 Hz and 60 Hz.

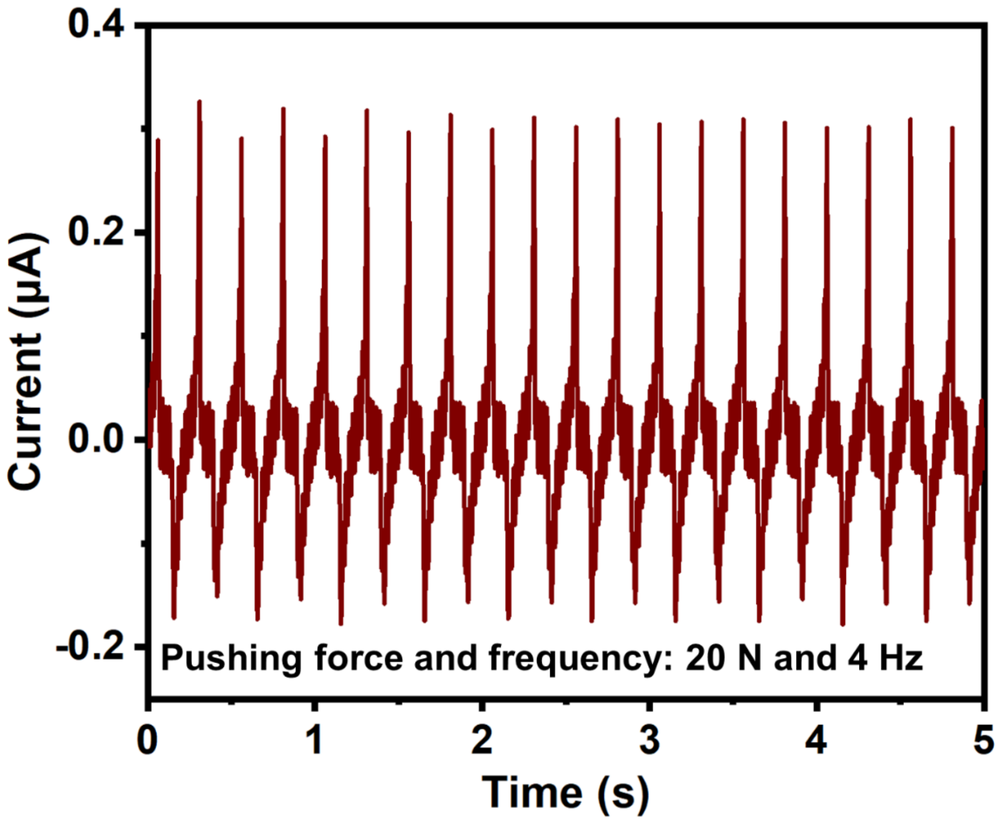

Supplementary Fig. 21 | The generated output current values of the (HaaOH)BF_4 device under 20 N pushing force.

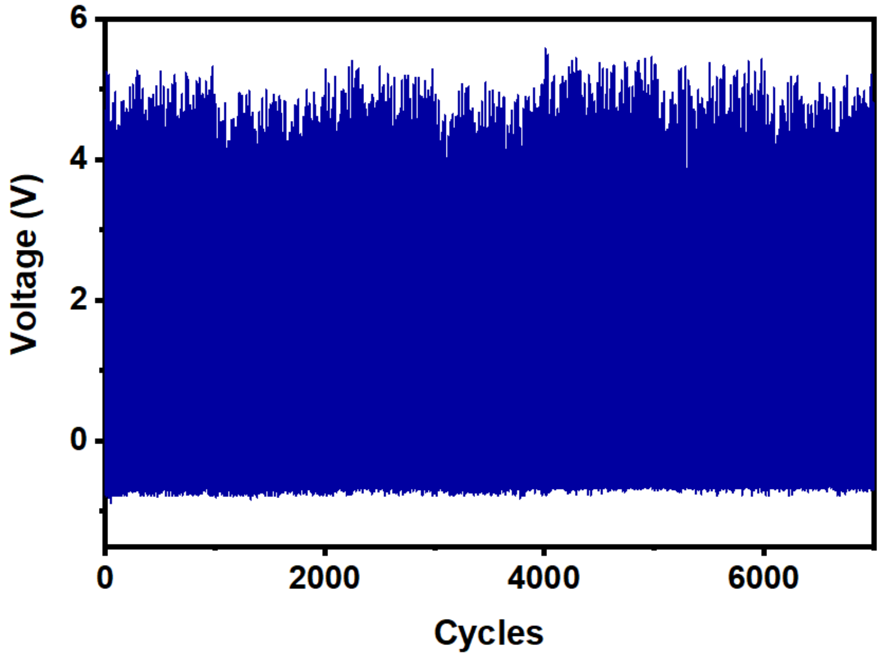

Supplementary Fig. 22 | Long-term sensing of the energy-harvesting device prepared using (HaaOH)BF_4 for more than 7000 cycles under 30 N impulse impact.

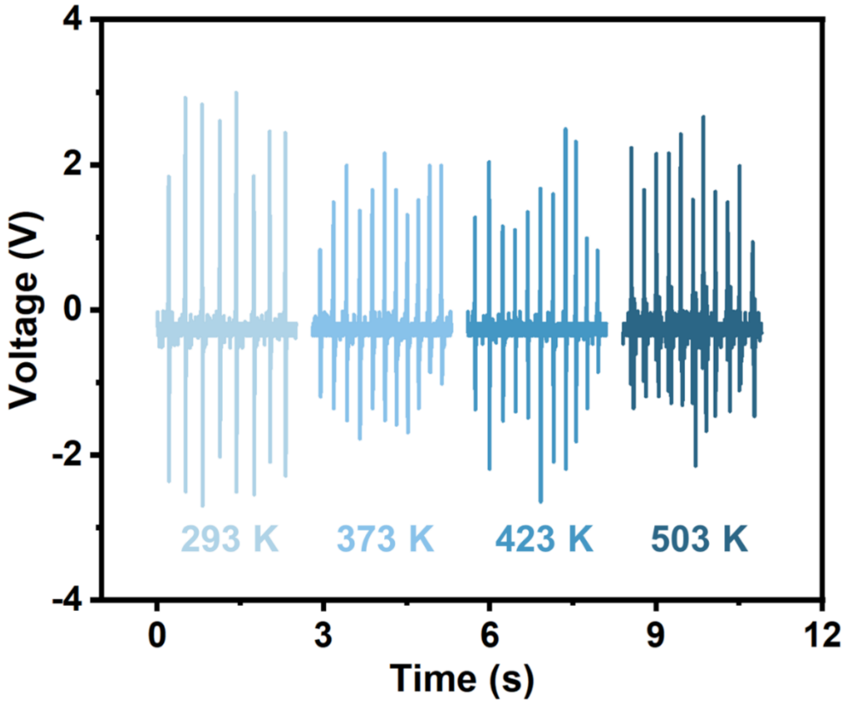

Supplementary Fig. 23 | The output signals of the (HaaOH)BF_4 piezoelectric device at different temperatures.

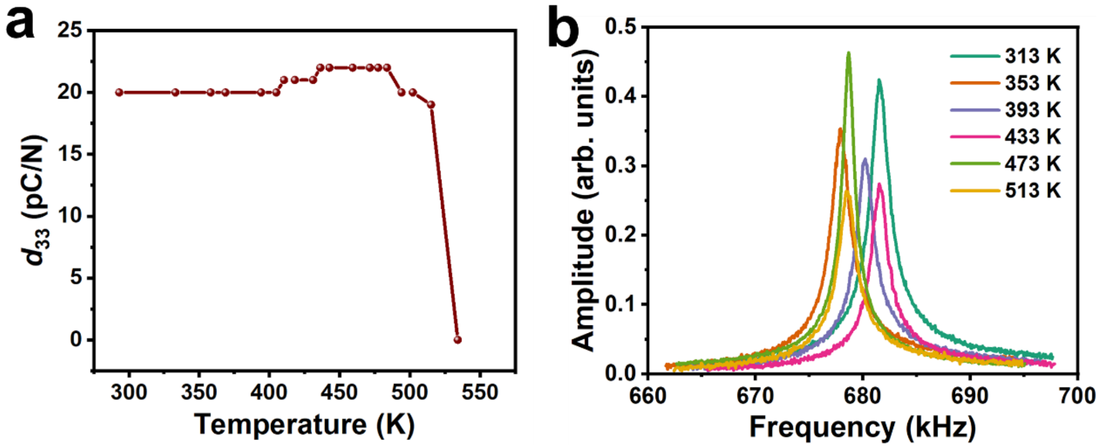

Supplementary Fig. 24 | Temperature-dependent piezoelectric properties of (HaaOH)BF_4. (a) Piezoelectric coefficient (d_{33}) of (HaaOH)BF_4 at different temperatures. (b) PFM resonance peaks at different temperatures.

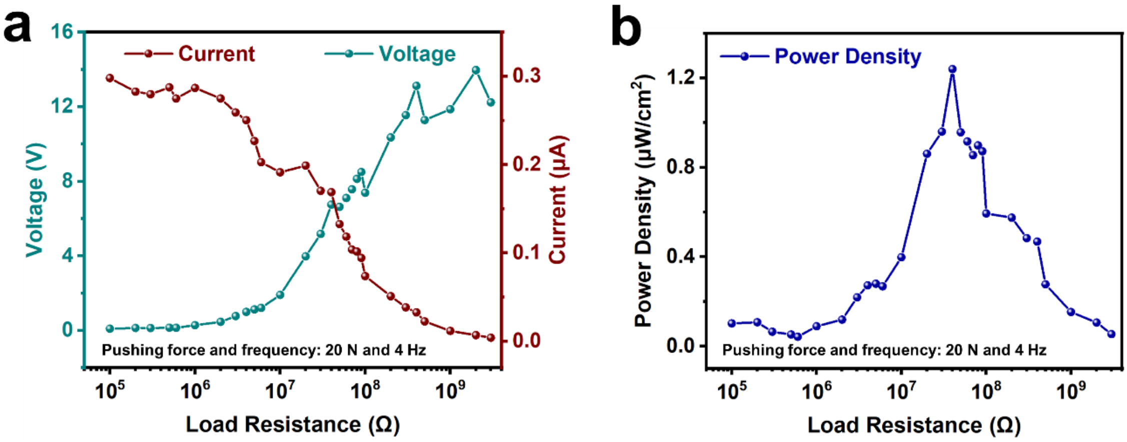

Supplementary Fig. 25 | The load resistance dependent measurements. (a) The load resistance dependent output voltage and current values and (b) output power density measured at various external load resistances values ranging from 1×10^5 to 3×10^9 Ω.

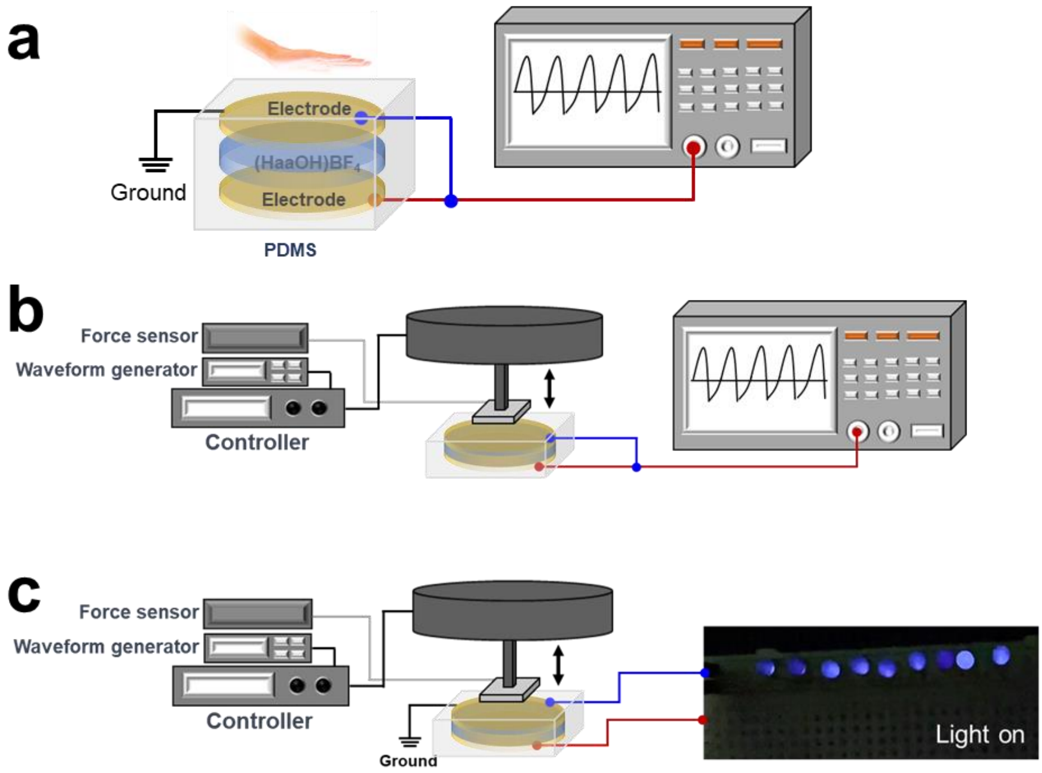

Supplementary Fig. 26 | Schematic diagram of device structure and testing system configuration. (a) Device structure of (HaaOH)BF_4 and sensor testing circuit. (b) Testing system configuration for piezoelectric devices. (c) Testing system configuration for lighting up the LEDs with mechanical pressure.

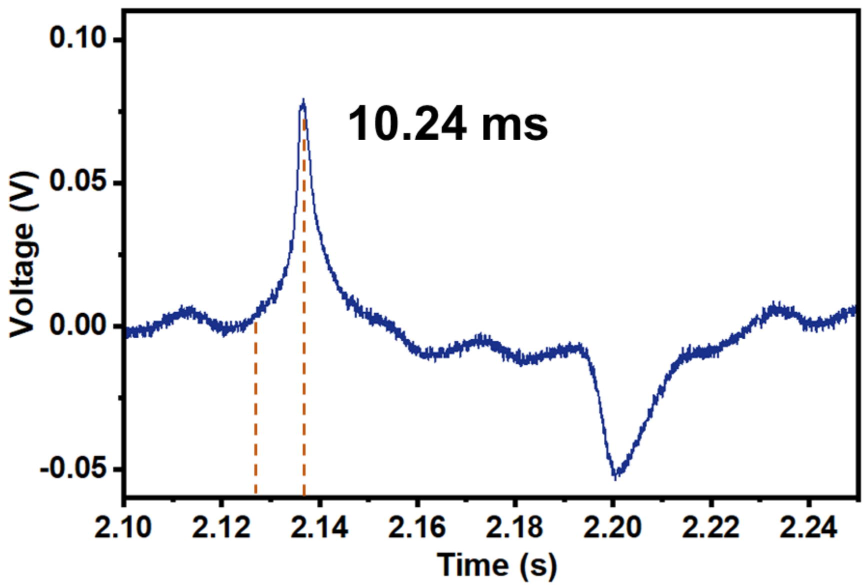

Supplementary Fig. 27 | The piezoelectric sensor shows a response time of 10.24 ms under tapping with the finger.

**Supplementary Table 1 | ** Summary of enhancing phase transition temperature in molecular ferroelectrics through various methods.

Prototype	T_1 (K)	Modified Compound	T_2 (K)	Modified Method	ΔT (K)	Ref.
$(Haa)BF_4$	192	$(HaaOH)BF_4$	528	Hydrogen Bond Modification	336	/
$[(MeO–C_6H_4–NH_3)(18\text{-crown-6})][BF_4]$	127	$[(MeO–C_6H_4–NH_3)(18\text{-crown-6})][TFSA]$	415	Momentum Matching	288	11
$[ABCH]CdCl_3$	190	$[4\text{-FABCH}]\text{-}CdCl_3$	419	H/F Substitution	229	12
$(PD)PbI_3$	260	$[4,4\text{-DFPD}]_2PbI_4$	429	H/F Substitution	169	13
CM-iodized salt	324	CM-chloride salt	453	Steric Confinement Modulation	129	14
$[H_2mdap]BiI_5$	264	$[H_2mdap]BiCl_5$	377	Steric Confinement Modulation	113	15
$[N\text{-MeDABCO}]PbI_3$	363	$[N\text{-FMedabco}]PbI_3$	473	H/F Substitution	110	13
$(Pyrrolidinium)CdCl_3$	240	(*R*)- and (*S*)-3-F-$(Pyrrolidinium)CdCl_3$	303	H/F substitution	63	16
[HDABCO][TFSA]	274	[DDABCO][TFSA]	327	Deuterium Isotope Effect	53	17
$(IBA)_2(EA)Pb_2Br_7$	326	$(IBA)_2(EA)_2Pb_3Br_{10}$	370	Cage-Confined Ethylamine Rotators	44	18
$(Pyrrolidinium)MnCl_3$	295	(*R*)- and (*S*)-3-(Fluoropyrrolidinium) $MnCl_3$	333	H/F Substitution	38	19
$(Benzylammonium)_2 PbBr_4$	405	$(Perfluorobenzylammonium)_2PbBr_4$	440	H/F Substitution	35	20
$[Me_3NCH_2Cl]CdBrCl_2$	373	$[Me_3NCH_2Cl]CdCl_3$	399	Steric Confinement Modulation	26	21

$MeO–C_6H_4–NH_3$ = 4-Methoxyanilinium; TFSA = Bis(trifluoromethanesulfonyl)ammonium; ABCH = 1-Azabicyclo[2.2.1]heptane; 4-FABCH = 4-Fluoro-1-Azabicyclo[2.2.1]heptane; PD = Piperidinium; 4,4-DFPD = 4,4-Difluoropiperidinium; CM = Cyclohexylmethylammonium; H_2mdap = N-Methyl-1,3-Propanediamine; N-Medabco = Methyldabconium; N-FMedabco = N-

Fluoromethyldabconium; DABCO = 1,4-Diazabicyclo[2.2.2] octane; IBA = Isobutylammonium; EA = Ethylammonium; Me = Methyl

Supplementary Table 2 | Crystal l data and structure refinement for $(HaaOH)BF_4$ at 293 K and $(Haa)BF_4$ at 293 K, respectively.

**Compound**	$\mathbf{(HaaOH)BF_4}$	$\mathbf{(Haa)BF_4}$	$\mathbf{(Haa)BF_4}$
Formula	$C_{10}H_{18}NOBF_4$	$C_{10}H_{18}NBF_4$	$C_{10}H_{18}NBF_4$
CCDC	2192545	2192429	2314039
Formula weight	255.06	239.06	239.06
Temperature/K	293	293	163
Crystal system	Orthorhombic	Orthorhombic	Monoclinic
Space group	$Pna2_1$	$Pnma$	$P2_1/c$
a/Å	13.0832(10)	22.9442(18)	23.9496(8)
b/Å	11.8671(10)	6.6697(6)	6.6354(2)
c/Å	8.1958(9)	7.8397(6)	15.3647(5)
α/deg	90	90	90
β/deg	90	90	108.7100(10)
γ/deg	90	90	90
Volume/Å^3	1272.5(2)	1199.72(17)	2312.65(13)
Z	4	4	8
Final R indices $R1[I > 2\sigma(I)]$	0.0798	0.0799	0.0502
$wR2[I > 2\sigma(I)]$	0.2622	0.2887	0.1533
Goodness-of-fit on F^2	1.018	1.105	1.065

Supplementary Table 3 | Hydrogen bond metrics of $(HaaOH)BF_4$ crystal

D-H…A	*d*(D-H)/Å	*d*(H…A)/Å	*d*(D…A)/Å	∠(D-H…A)/°
N1-H1B…O1	0.890	1.922	2.801	168.6
N1-H1A…F1C	0.891	2.073	2.961	174.2
N1-H1C…F1B	0.891	2.086	2.931	158.0
O1-H1…F1A	0.820	2.004	2.812	168.3

Supplementary References

1. King-Smith RD, Vanderbilt D. Theory of polarization of crystalline solids. *Physical Review B* **47**, 1651-1654 (1993).

2. Vanderbilt D, King-Smith RD. Electric polarization as a bulk quantity and its relation to surface charge. *Physical Review B* **48**, 4442-4455 (1993).

3. Kresse G, Furthmüller J. Efficient iterative schemes for ab initio total-energy calculations using a plane-wave basis set. *Physical Review B* **54**, 11169-11186 (1996).

4. Kresse G, Furthmüller J. Efficiency of ab-initio total energy calculations for metals and semiconductors using a plane-wave basis set. *Computational Materials Science* **6**, 15-50 (1996).

5. Perdew JP, Burke K, Ernzerhof M. Generalized Gradient Approximation Made Simple. *Physical Review Letters* **77**, 3865-3868 (1996).

6. Spackman MA, Byrom PG. A novel definition of a molecule in a crystal. *Chemical Physics Letters* **267**, 215-220 (1997).

7. Spackman MA, Jayatilaka D. Hirshfeld surface analysis. *CrystEngComm* **11**, 19-32 (2009).

8. Wood PA, McKinnon JJ, Parsons S, Pidcock E, Spackman MA. Analysis of the compression of molecular crystal structures using Hirshfeld surfaces. *CrystEngComm* **10**, 368-376 (2008).

9. Turner MJ, Grabowsky S, Jayatilaka D, Spackman MA. Accurate and Efficient Model Energies for Exploring Intermolecular Interactions in Molecular Crystals. *The*

Journal of Physical Chemistry Letters **5**, 4249-4255 (2014).

10. Turner MJ, Thomas SP, Shi MW, Jayatilaka D, Spackman MA. Energy frameworks: insights into interaction anisotropy and the mechanical properties of molecular crystals. *Chemical Communications* **51**, 3735-3738 (2015).

11. Song X-J*, et al.* Record Enhancement of Curie Temperature in Host–Guest Inclusion Ferroelectrics. *Journal of the American Chemical Society* **143**, 5091-5098 (2021).

12. Tang Y-Y*, et al.* Record Enhancement of Phase Transition Temperature Realized by H/F Substitution. *Advanced Materials* **32**, 2003530 (2020).

13. Ai Y, Lv H-P, Wang Z-X, Liao W-Q, Xiong R-G. H/F substitution for advanced molecular ferroelectrics. *Trends in Chemistry* **3**, 1088-1099 (2021).

14. Xu H*, et al.* Record high-T_c and large practical utilization level of electric polarization in metal-free molecular antiferroelectric solid solutions. *Nature Communications* **13**, 5329 (2022).

15. Chen Q*, et al.* High-Tc Realization of Lead-Free Halide Hybrid Ferroelectrics via Steric Confinement Modulation. *Advanced Functional Materials* **33**, 2213964 (2023).

16. Tang Y-Y, Ai Y, Liao W-Q, Li P-F, Wang Z-X, Xiong R-G. H/F-Substitution-Induced Homochirality for Designing High-T_c Molecular Perovskite Ferroelectrics. *Advanced Materials* **31**, 1902163 (2019).

17. Song X-J*, et al.* Bistable State of Protons for Low-Voltage Memories. *Journal of the American Chemical Society* **142**, 9000-9006 (2020).

18. Peng Y*, et al.* Acquiring High-T_C Layered Metal Halide Ferroelectrics via Cage-Confined Ethylamine Rotators. *Angewandte Chemie International Edition* **60**, 2839-2843 (2021).

19. Ai Y*, et al.* Fluorine Substitution Induced High T_c of Enantiomeric Perovskite Ferroelectrics: (*R*)- and (*S*)-3-(Fluoropyrrolidinium)$MnCl_3$. *Journal of the American Chemical Society* **141**, 4474-4479 (2019).

20. Zhang H-Y, Zhang Z-X, Song X-J, Chen X-G, Xiong R-G. Two-Dimensional Hybrid Perovskite Ferroelectric Induced by Perfluorinated Substitution. *Journal of the American Chemical Society* **142**, 20208-20215 (2020).

21. Chen X-G, *et al*. Remarkable Enhancement of Piezoelectric Performance by Heavy Halogen Substitution in Hybrid Perovskite Ferroelectrics. *Journal of the American Chemical Society* **145**, 1936-1944 (2023).
