# Supplementary material for: Enhancement of phase transition temperature through hydrogen bond modification in molecular ferroelectrics
Source: Nat Commun. 2024 May 25;15:4470. doi: 10.1038/s41467-024-48948-0 (PMC11127950; doi:10.1038/s41467-024-48948-0)
Supplement: Supplementary file 3 — Description of Additional Supplementary Files [file 41467_2024_48948_MOESM3_ESM.pdf]

Description of Additional Supplementary Files

Supplementary Movie 1. Illuminate LEDs through mechanical tapping (HaaOH)BF_4 device.

Supplementary Movie 2. Signals of the output voltage in the process of light, normal and heavy tapping the (HaaOH)BF_4 device on a dummy.
