# Supplementary material for: Enhancement of phase transition temperature through hydrogen bond modification in molecular ferroelectrics
Source: Nat Commun. 2024 May 25;15:4470. doi: 10.1038/s41467-024-48948-0 (PMC11127950; doi:10.1038/s41467-024-48948-0)
Supplement: Supplementary file 6 — Reporting Summary [file 41467_2024_48948_MOESM6_ESM.pdf]

nature portfolio

Corresponding author(s): Yu-Meng You; Jun Wu

Last updated by author(s): May 6, 2024

# Reporting Summary

Nature Portfolio wishes to improve the reproducibility of the work that we publish. This form provides structure for consistency and transparency in reporting. For further information on Nature Portfolio policies, see our Editorial Policies and the Editorial Policy Checklist.

## Statistics

For all statistical analyses, confirm that the following items are present in the figure legend, table legend, main text, or Methods section.

| n/a | Confirmed | |
|---|---|---|
| ☐ | ☒ | The exact sample size (*n*) for each experimental group/condition, given as a discrete number and unit of measurement |
| ☐ | ☒ | A statement on whether measurements were taken from distinct samples or whether the same sample was measured repeatedly |
| ☐ | ☒ | The statistical test(s) used AND whether they are one- or two-sided<br>*Only common tests should be described solely by name; describe more complex techniques in the Methods section.* |
| ☒ | ☐ | A description of all covariates tested |
| ☒ | ☐ | A description of any assumptions or corrections, such as tests of normality and adjustment for multiple comparisons |
| ☐ | ☒ | A full description of the statistical parameters including central tendency (e.g. means) or other basic estimates (e.g. regression coefficient) AND variation (e.g. standard deviation) or associated estimates of uncertainty (e.g. confidence intervals) |
| ☒ | ☐ | For null hypothesis testing, the test statistic (e.g. *F*, *t*, *r*) with confidence intervals, effect sizes, degrees of freedom and *P* value noted<br>*Give P values as exact values whenever suitable.* |
| ☒ | ☐ | For Bayesian analysis, information on the choice of priors and Markov chain Monte Carlo settings |
| ☒ | ☐ | For hierarchical and complex designs, identification of the appropriate level for tests and full reporting of outcomes |
| ☒ | ☐ | Estimates of effect sizes (e.g. Cohen's *d*, Pearson's *r*), indicating how they were calculated |

*Our web collection on statistics for biologists contains articles on many of the points above.*

## Software and code

Policy information about availability of computer code

| | |
|---|---|
| Data collection | No software was used |
| Data analysis | No software was used |

For manuscripts utilizing custom algorithms or software that are central to the research but not yet described in published literature, software must be made available to editors and reviewers. We strongly encourage code deposition in a community repository (e.g. GitHub). See the Nature Portfolio guidelines for submitting code & software for further information.

## Data

Policy information about availability of data

All manuscripts must include a data availability statement. This statement should provide the following information, where applicable:

- Accession codes, unique identifiers, or web links for publicly available datasets
- A description of any restrictions on data availability
- For clinical datasets or third party data, please ensure that the statement adheres to our policy

All data generated and analyzed in this study are included in the Article and its Supplementary Information. And all relevant data that support the findings of this study are available from the corresponding authors upon request.
The crystal structures generated in this study have been deposited in the Cambridge Crystallographic Data Centre under accession code CCDC: 2192545, 2192429 and 2314039. The data can be obtained free of charge via www.ccdc.cam.ac.uk/data_request/cif, or by emailing data_request@ccdc.cam.ac.uk, or by contacting

The Cambridge Crystallographic Data Centre, 12 Union Road, Cambridge CB2 1EZ, UK; fax: +44 1223 336033.

## Research involving human participants, their data, or biological material

Policy information about studies with human participants or human data. See also policy information about sex, gender (identity/presentation), and sexual orientation and race, ethnicity and racism.

| | |
|---|---|
| Reporting on sex and gender | N/A |
| Reporting on race, ethnicity, or other socially relevant groupings | N/A |
| Population characteristics | N/A |
| Recruitment | N/A |
| Ethics oversight | N/A |

Note that full information on the approval of the study protocol must also be provided in the manuscript.

# Field-specific reporting

Please select the one below that is the best fit for your research. If you are not sure, read the appropriate sections before making your selection.

☐ Life sciences ☐ Behavioural & social sciences ☒ Ecological, evolutionary & environmental sciences

For a reference copy of the document with all sections, see nature.com/documents/nr-reporting-summary-flat.pdf

# Ecological, evolutionary & environmental sciences study design

All studies must disclose on these points even when the disclosure is negative.

| | |
|---|---|
| Study description | We present a hydrogen bond modification method by introducing the hydroxyl group, and successfully introduce ferroelectric polarization and boost the phase transition temperature by at least 336 K in the molecular ferroelectric 1-hydroxy-3-adamantanammonium tetrafluoroborate. And we explore the piezoelectric sensor devices of the non-metallic molecular ferroelectric. |
| Research sample | 1-hydroxy-3-adamantanammonium tetrafluoroborate (HaaOH)BF4; 1-adamantanammonium tetrafluoroborate (Haa)BF4 |
| Sampling strategy | The single crystals of (HaaOH)BF4 and (Haa)BF4 were used in the article that were obtained by slowly evaporating the deionized water solutions at room temperature. |
| Data collection | Y.-A. X prepared the samples and performed the PFM measurements, piezoelectric energy-harvesting devices and analysis. S.-S. D prepared and test the power supply and stimuli sensing of piezoelectric devices. H.-H. H measured the thermodynamic properties and sorted out the data. J. Y performed the single crystal measurement and analysis. Q. P contributed to P-V loop measurements. T.-T. S, X. W and H.-R. J participated in the production and performance testing of piezoelectric devices. Y.-M. Y and J. W conceived and supervised the study. |
| Timing and spatial scale | Data collection for this article began in August 2022 and ended in March 2024. The crystal samples are firstly synthesized, and then their crystal structures and physical properties were characterized, including phase transition, ferroelectric and piezoelectric properties. Each characterization experiment process involves many repetitions, so this step takes the longest time, is complex and has no specific sequence. |
| Data exclusions | No data were excluded from the analyses. |
| Reproducibility | All attempts to repeat the experiment were successful. |
| Randomization | The randomization is not relevant to our study, because the structure of the crystal samples obtained by repeated synthesis is determined, and the purity can reach 100%. |
| Blinding | We have arranged for two authors to conduct experiments independently, characterizing samples and collecting data at different times, to ensure that the final results are consistent. |

Did the study involve field work? ☐ Yes ☒ No

# Reporting for specific materials, systems and methods

We require information from authors about some types of materials, experimental systems and methods used in many studies. Here, indicate whether each material, system or method listed is relevant to your study. If you are not sure if a list item applies to your research, read the appropriate section before selecting a response.

## Materials & experimental systems

| n/a | Involved in the study |
|---|---|
| ☒ | ☐ Antibodies |
| ☒ | ☐ Eukaryotic cell lines |
| ☒ | ☐ Palaeontology and archaeology |
| ☒ | ☐ Animals and other organisms |
| ☒ | ☐ Clinical data |
| ☒ | ☐ Dual use research of concern |
| ☒ | ☐ Plants |

## Methods

| n/a | Involved in the study |
|---|---|
| ☒ | ☐ ChIP-seq |
| ☒ | ☐ Flow cytometry |
| ☒ | ☐ MRI-based neuroimaging |

# Plants

| | |
|---|---|
| Seed stocks | N/A |
| Novel plant genotypes | N/A |
| Authentication | N/A |
